# Ankle MRI and preceding radiographs: an evaluation of physician ordering practices

**DOI:** 10.1007/s00256-022-04084-8

**Published:** 2022-06-06

**Authors:** Kristopher de Ga, Dylan Noblett, Cyrus Bateni

**Affiliations:** Department of Radiology, University of California Davis Medical Center, 4860 Y Street, Suite 3100, Sacramento, CA 95817 USA

**Keywords:** Ankle, MRI, Radiography, Imaging utilization

## Abstract

**Objective:**

Multiple guidelines have been published for appropriate imaging in patients with ankle-related symptoms which suggest radiographs as the initial imaging examination for both acute and chronic ankle abnormalities. Few studies have evaluated adherence to these imaging guidelines. This study retrospectively evaluated the utilization of ankle MRI and preceding radiographs based on ordering provider group and MRI indication.

**Materials and methods:**

A total of 4186 ankle MRIs performed over a 9-year period at a single institution were evaluated for the presence of preceding ankle and/or foot radiographs at two time points, within 3 months and within 6 months of the MRI examination. Ankle MRIs were then categorized based on 6 ordering provider groups and 13 MRI indications.

**Results:**

Of the 4186 MRIs evaluated, 68% had preceding radiographs within 3 months and 77% had radiographs within 6 months. Primary care, sports medicine, and podiatry had the lowest rates of preceding radiographs (73%, 68%, and 64%, respectively, within 6 months). Eighty-six percent of ankle MRIs ordered by orthopedic surgery had preceding radiographs within 6 months and 89% of ankle MRIs ordered by emergency medicine and inpatient providers had preceding radiographs. MRIs intended for evaluation of Achilles tendon or plantar fascia abnormalities were among the least likely indications to have preceding radiographs.

**Conclusion:**

Based on established clinical guidelines, there was a lower-than-expected rate of obtaining preceding radiographs for ankle MRIs among most provider groups, particularly non-orthopedic outpatient providers. Additional research is needed to address the lack of adherence to clinical imaging guidelines and ensure appropriate imaging.

## Introduction

Musculoskeletal disorders of the ankle are a common cause of disability and pain, with an estimated prevalence of ankle pain in the general adult population of 9 to 15% [1]. Patients with ankle-related symptoms often present to a wide range of specialties, including primary care, orthopedic, and emergency medicine providers. Following history and physical examination, imaging is commonly employed to assist in the diagnosis and management of ankle symptomatology.

Beginning with the Ottawa ankle rules in 1996, clinicians have been provided multiple resources on appropriate imaging in patients with ankle-related symptoms [2]. In 2000, the American College of Radiology (ACR) published Appropriateness Criteria for imaging acute and chronic ankle symptoms, recommending ankle radiographs as the initial imaging exam for both acute and chronic ankle symptoms [3, 4]. These guidelines rate ankle MRI as a “usually not appropriate” initial test and reserve MRI for patients requiring further evaluation following radiographs. Similarly, the Ottawa ankle rules and the more recent 2018 Choosing Wisely recommendations from the Pediatric Orthopedic Society of North America suggest radiography as the initial best test when imaging is indicated [5]. The rationale is that radiographs may provide all the diagnostic imaging information needed for appropriate management, potentially eliminating the need for more costly subsequent imaging. Preceding radiographs are often useful correlates when interpreting MRI, because findings such as small ossific fragments, calcifications, and osseous matrix may be more apparent on radiography [6]. MRI may be helpful in a variety of circumstances, but generally only after initial radiographs are performed.

Few studies have examined the adherence to appropriate imaging algorithms in clinical practice for musculoskeletal conditions. One study focused on quantifying adherence to the ACR Appropriateness Criteria for nontraumatic knee pain found 43% of knee MRIs ordered at their institution had a “usually not appropriate” rating [7]. A small retrospective study by Gross in 2017 suggested significant lack of adherence to accepted imaging algorithms, with only 67.9% of ankle MRIs performed with preceding radiographs [8]. A larger study focused on knee and shoulder MRI utilization in the Medicare population found only 72% of knee MRIs and 63–65% of shoulder MRIs were performed with recent radiographs [9]. They estimated the expense of these potentially unwarranted MRIs to be between $20 and 35 million.

The purpose of this study was to retrospectively evaluate the utilization of ankle MRI and preceding radiographs based on physician ordering group and MRI indication. We hypothesized that a substantial percentage of ankle MRIs were obtained without preceding ankle or foot radiographs, and therefore discordant with appropriate imaging guidelines, including those from the ACR.

## Methods

### Design and patient criteria

Our institutional review board approved a retrospective review of non-contrast ankle MRIs and associated radiograph reports performed at a single large academic health center from 2011 to 2019. The requirement to obtain consent was waived. We used a radiology data-mining system (Montage; Montage Healthcare Systems, Philadelphia, PA, USA) to identify all non-contrast ankle MRIs performed between January 1, 2011, and December 31, 2019, excluding prisoners and patients ≤ 5 years of age. Patients ≤ 5 years of age were excluded as they are specifically excluded from the ACR Appropriateness Criteria for ankle imaging.

Ankle MRIs were assessed for the presence or absence of preceding radiographs, including those of the ankle, calcaneus, or foot, initially using the radiology data-mining system followed by review of the imaging archive system and electronic medical record. Preceding radiographs were evaluated at two time points, within 3 months and within 6 months of the ankle MRI examination. Individual electronic medical records were also reviewed for documentation of preceding radiographs performed at outside facilities. Ankle MRIs were excluded from our analysis in cases where it was unknown or unclear if preceding radiographs were performed. This largely included outside referrals that lacked documentation in our electronic medical record.

A board-certified musculoskeletal radiologist with 9 years of experience and two radiology residents then analyzed the radiology reports to categorize the physician ordering groups and provided MRI indications, with all exams evaluated by at least two reviewers.

Ordering providers were categorized under 6 different groups—primary care (internal medicine, family medicine, and pediatric clinicians), orthopedic surgery, sports medicine (non-surgical), podiatry, inpatient/emergency medicine providers, and others not fitting into these categories (neurology, rheumatology, etc.).

MRI indications were classified under 12 different categories based on the primary ankle MRI indications outlined in the practice parameters collaboratively developed by the American College of Radiology, Society of Pediatric Radiology, and Society of Skeletal Radiology [10]. Each MRI examination was designated a maximum of two indications. For exams that did not fit easily into these 12 categories, such as a provided indication of nonspecific pain, we created a category of “unexplained pain/swelling or other etiologies.” Due to their infrequency, this category also included very uncommon indications evaluating congenital, neurologic, and synovial conditions.

### Statistical analysis

Proportions of categorical variables were compared with use of the Pearson chi-square test. Means were compared with two-tailed, unpaired *t* tests. In the comparison of MRI indication and presence of preceding radiographs, individual chi-square tests were performed for each MRI indication. All statistical calculations were performed with use of Stata 16 (StataCorp, College Station, TX) with the level of significance set at *p* < 0.05. Ninety-five percent confidence intervals were calculated for each provider group.

## Results

A total of 4343 MRIs were performed at our institution over the 9-year period. One hundred fifty-seven of these examinations were excluded from the analysis due to uncertainty of preceding radiographs following review of the medical record and imaging archive system. Of the remaining 4186 qualifying examinations, 2865 (68%) had preceding radiographs within 3 months of the ankle MRI and 3209 (77%) had preceding radiographs within 6 months.

**Fig. 1:**
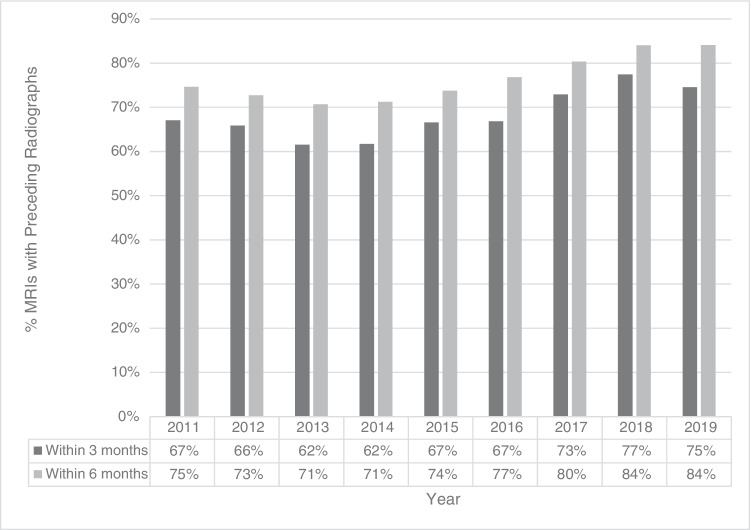
MRI examinations over time according to presence of preceding radiographs

The radiographs group at 3 months had an average age of 45.1 years, while the no radiographs group had an average age of 45.9 years (Table 1). The radiographs group at 6 months had an average age of 45.2 years and the no radiographs group had an average age of 45.3 years (Table 2). Sex did not significantly differ between the radiographs and no radiographs groups at either time point. A total of 59.6% of all patients were female and the average age in years for all patients was 45.3 +/− 17.7 (Table 1). Overall, MRI utilization increased over time, and, in more recent years (2017–2019), a higher proportion of MRIs was performed with preceding radiographs for both time points (Fig. 1).

**Table 1:** Comparison of patient demographics according to presence of preceding radiographs within 3 months

	Radiographs (*N* = 2865)	No radiographs (*N* = 1321)	Total (*N* = 4186)	*p*-value
Sex				0.65
Female	59.9% (1717)	59.2% (782)	59.7% (2499)	
Male	40.1% (1148)	40.8% (539)	40.3% (1687)	
Mean age +/− std dev (yr.)	45.1 +/− 17.7	45.9 +/− 17.5	45.3 +/− 17.7	0.16

**Table 2:** Comparison of patient demographics according to presence of preceding radiographs within 6 months

	Radiographs (*N* = 3209)	No radiographs (*N* = 977)	Total (*N* = 4186)	*p*-value
Sex				0.90
Female	59.6% (1914)	59.9% (585)	59.7% (2499)	
Male	40.4% (1295)	40.1% (392)	40.3% (1687)	
Mean age+/− std dev (yr.)	45.2 +/− 17.7	45.7 +/− 17.5	45.3 +/− 17.7	0.44

The majority of all ankle MRIs were ordered by primary care and orthopedic surgery, 42% and 37%, respectively (Table 3). Primary care, sports medicine, podiatry, and “other” providers all obtained radiographs less than 75% of the time prior to ordering ankle MRIs at both time points (67%, 55%, 52%, and 56%, respectively, within 3 months and 73%, 68%, 64%, and 70%, respectively, within 6 months). Inpatient and emergency department providers ordering ankle MRIs had preceding radiographs for 89% of patients at the 3-month and 6-month time points. When looking at orthopedic surgery, there were preceding radiographs for 77% of ankle MRIs within 3 months of the exam and 86% within 6 months. Among all non-orthopedic providers combined, 63% of ankle MRIs had preceding radiographs within 3 months (*p* < 0.01) and 71% within 6 months (*p* < 0.01).

**Table 3:** Comparison within ordering groups according to presence of preceding radiographs

Ordering group	Radiographs within 3 months % within row 95% *CI*	Radiographs within 6 months % within row 95% *CI*
Primary care (*N* = 1742)	67%	73%
	64–69%	71–75%
Orthopedic surgery (*N* = 1530)	77%	86%
	75–80%	84–87%
Sports medicine (*N* = 456)	55%	68%
	51–60%	64–73%
Podiatry (*N* = 306)	52%	64%
	46–58%	58–69%
Inpatient/emergency medicine (*N* = 65)	89%	89%
	79–96%	79–96%
Other (*N* = 87)	56%	70%
	45–67%	59–79%
Total (*N* = 4186)	68%	77%
	67–70%	75–78%

The most common ankle MRI indications were for the evaluation of “ligament injury, sprain, or instability,” “marrow abnormalities, fracture, or acute trauma,” “Achilles tendon abnormalities,” and “unexplained pain/swelling or other etiologies” (Table 4). Only 45% of MRIs ordered for the evaluation of the plantar fascia had preceding radiographs within 3 months, and 57% had radiographs within 6 months. Similarly, only 51% of MRIs ordered for the evaluation of Achilles tendon abnormalities had preceding radiographs within 3 months and 59% had radiographs within 6 months. Although no indications were significantly less likely to obtain preceding radiographs at either time point, most indications did not exceed 80% at 6 months.

**Table 4:** Comparison of MRI indication according to presence of preceding radiographs

MRI indication (% of all indications)	% Radiographs within 3 months (*p*-value)	% Radiographs within 6 months (*p*-value)
Marrow abnormalities, fracture, or acute trauma (11%)	82% (< 0.01)	87% (< 0.01)
Ligament injury, sprain, or instability (17%)	78% (< 0.01)	85% (< 0.01)
Osteochondral lesion (9%)	74% (< 0.01)	84% (< 0.01)
Peroneal tendon abnormality (5%)	70% (< 0.01)	80% (< 0.01)
Unexplained pain/swelling or other etiologies (22%)	69% (< 0.01)	78% (< 0.01)
Posterior tibialis tendon abnormality (6%)	69% (< 0.01)	77% (< 0.01)
Abnormality of other ankle tendons (3%)	69% (< 0.01)	75% (< 0.01)
Impingement (2%)	68% (< 0.01)	73% (< 0.01)
Tumor/infection (2%)	67% (< 0.01)	69% (< 0.01)
Arthritis or alignment abnormality (3%)	66% (< 0.01)	80% (< 0.01)
Pre-/post-operative evaluation (6%)	61% (< 0.01)	73% (< 0.01)
Achilles tendon abnormality (11%)	51% (0.61)	59% (< 0.01)
Plantar fascia abnormality (3%)	45% (0.27)	57% (0.08)

*Individual chi-square tests performed for each MRI indication at each time point

## Discussion

Our study demonstrated preceding radiographs were obtained for 77% of qualifying ankle MRIs within 6 months of the exam. The majority of ordering groups demonstrated relatively low rates of obtaining radiography initially, particularly among non-orthopedic providers. Inpatient and emergency medicine providers were the exception to this practice, having radiographs prior to MRI in 89% of cases. We believe this is due to the comparatively decreased availability of musculoskeletal MRI in the emergency and inpatient settings at our institution and the majority of emergency/inpatient care at our institution following a standardized trauma workflow starting with radiography.

This study was the largest evaluation of radiography prior to ankle MRI to our knowledge. Our rate of 77% of ankle MRIs performed with preceding radiographs is slightly higher than previous studies focused on musculoskeletal MRI of various joints, which have ranged from 60 to 72% [7–9, 11]. Many studies have reported their rates of obtaining radiographs by comparing non-orthopedic and orthopedic providers. In our study, correlating rates of obtaining radiographs within 6 months were 71% for non-orthopedic providers and 86% for orthopedic providers.

We chose to include patients who underwent either preceding ankle or foot radiographs as being compliant with national imaging guidelines. We wanted to ensure that we captured ankle MRIs ordered for the evaluation of conditions such as hindfoot arthritis, Achilles tendon symptoms, or plantar fascia abnormalities that might have preceding foot radiographs but not ankle radiographs. We believe foot radiographs are often an adequate initial evaluation of these conditions and dedicated ankle radiographs may not be warranted. Despite the inclusion of foot radiographs, MRIs intended for the evaluation of Achilles tendon and plantar fascia abnormalities had the lowest rates of obtaining preceding radiographs at both time points. This trend may be due to the perceived limited evaluation of these structures with radiography.

Ankle MRIs for the evaluation of “ligament injury, sprain, or instability,” “marrow abnormalities, fracture, or acute trauma,” osteochondral lesions, abnormalities of the peroneal and “other” ankle tendons, arthritis and alignment abnormalities, and “unexplained pain/swelling or other etiologies” were more likely to have preceding radiographs, all at rates greater than 80% within 6 months of the ankle MRI. While this is an improvement compared to other indications, we still consider this a relatively low rate of obtaining radiographs considering that many of these abnormalities can be adequately evaluated initially with radiographs, followed by advanced imaging such as CT or MRI if necessary.

When utilized appropriately, ankle MRI can make a dramatic impact on patient diagnosis and management [12–14]. However, we emphasize that radiographs remain the first recommended imaging modality in the evaluation of acute and chronic ankle symptoms as many diagnoses can be made with radiographs alone [3, 4]. In instances where an MRI is clinically warranted regardless of radiographic findings (such as primarily soft tissue injury), we maintain that radiographs may still assist in the interpretation of these MRIs.

Prior studies have demonstrated inappropriate use of imaging for musculoskeletal conditions and have suggested low adherence to imaging guidelines [7–9, 11, 15–22]. Several studies have suggested potential reasons for inappropriate imaging utilization, including level of provider experience, specialty type, low awareness of appropriate imaging recommendations, lack of emphasis in initial medical training, and increasing availability/demand of advanced imaging [23–27]. The increasing integration of imaging clinical decision support tools into electronic medical records may help improve appropriate imaging at the institutional level [28]. However, due to the multifactorial nature of this complex issue, further development of these support tools would ideally incorporate individual patient characteristics into the imaging recommendations [29].

Our study evaluated for the presence of preceding radiographs at two time points, within 3 months and within 6 months of the ankle MRI. To our knowledge, no established guidelines comment on the optimal timing for preceding radiographs. Previous studies focused on preceding radiography for musculoskeletal MRI of various joints have varied widely on this topic. One study evaluated for radiographs within 90 days, two studies evaluated for radiographs within 6 months, and one study evaluated for radiographs within 12 months [7–9, 11]. While the optimal timing of preceding radiography will depend on the individual clinical scenario, an established guideline may be helpful for both ordering providers and radiologists to determine when updated radiographs should be obtained prior to MRI.

We recognize our study has limitations. While we attempted to account for patients who underwent imaging at outside institutions, we ultimately excluded 157 ankle MRIs where it was unclear when or if preceding radiographs were performed due to insufficient available documentation. This represented a small proportion of the examinations at less than 4% and likely would not significantly alter our results. As a single large academic center, the MRI ordering practices we encountered may not be reflective of practice in different clinical settings. Our study spanned across multiple revisions of the ACR Appropriateness Criteria, as there were minor revisions of the acute imaging guidelines in 2015 and of the chronic imaging guidelines in 2018. Despite these revisions, however, these guidelines have maintained that ankle radiographs are the best initial imaging exam. In addition, while the Choosing Wisely recommendations have been in place since 2012, specific guidelines pertaining to radiography and MRI of the ankle were not published until early 2018.

Our study did not attempt to analyze the findings of the ankle MRIs or determine whether the findings affected clinical management, as this was beyond the scope of our hypothesis. Further research in this area may assist in refining physician ordering guidelines and determining which ankle MRI indications may or may not benefit from preceding radiographs and/or ankle MRI.

As the utilization of MRI increases, we must ensure that we recommend and perform appropriate imaging. Imaging guidelines supported by national organizations including Choosing Wisely and the ACR Appropriateness Criteria should be a commonly used resource for radiologists and ordering physicians. Further research is needed to determine effective interventions for improving appropriate imaging utilization.
